# Cardiac *Candida krusei* infection

**DOI:** 10.4103/0974-2069.64355

**Published:** 2010

**Authors:** Viroj Wiwanitkit

**Affiliations:** Wiwanitkit House, Bangkhae, Bangkok, Thailand. E-mail: wviroj@yahoo.com

Sir,

I read the report by Patted *et al*. on cardiac *Candida krusei* infection presenting as a right ventricular mass with great interest.[1] Patted *et al*. have mentioned that necrotizing enterocolitis and disseminated fungal infection result in an adverse outcome as was seen in their case.[1] Indeed, *Candida krusei* infection is difficult to treat. Majority of the reported cases with hematogenous spread have died[2] Basic antifungal treatment such as Amphotericin B administration is usually unsuccessful.[3] In the case of cardiac infection, cardiac ischemia leading to death has also been reported.[2] Intravenous Caspofungin therapy has been reported to be the only successful treatment for this condition.[4]
